# Safety in Spine Surgery: Risk Factors for Intraoperative Blood Loss and Management Strategies

**DOI:** 10.3390/life15101615

**Published:** 2025-10-16

**Authors:** Magdalena Rybaczek, Piotr Kowalski, Zenon Mariak, Michał Grabala, Joanna Suszczyńska, Tomasz Łysoń, Paweł Grabala

**Affiliations:** 1 Department of Neurosurgery, Medical University of Bialystok, M. Sklodowskiej-Curie 24A, 15-276 Bialystok, Poland; magdalenarybaczek@interia.pl (M.R.); zenon.mariak@umb.edu.pl (Z.M.); lyson_t@vp.pl (T.Ł.); 2 Department of Neurosurgery, Regional Specialized Hospital, ul. Dekerta 1, 66-400 Gorzow, Poland; pkowal72@gmail.com; 3 2nd Clinical Department of General and Gastroenterogical Surgery, Medical University of Bialystok, M. Skłodowskiej-Curie 24A, 15-276 Bialystok, Poland; michal@grabala.pl (M.G.); joanna.suszczynska@uskwb.pl (J.S.); 4 Department of Neurosurgery, Polish-Mother’s Memorial Hospital Research Institute, Rzgowska 281/289, 93-338 Lodz, Poland

**Keywords:** massive blood loss, spine surgery, tranexamic acid, anticoagulants, antiplatelets, hemostasis, transfusion, risk factors, NOACs, perioperative management

## Abstract

Background: Massive intraoperative blood loss (IBL) is a serious complication in complex spine surgeries such as deformity correction, multilevel fusion, tumor resection, and revision procedures. While no strict definition exists, blood loss exceeding 1500 mL or 20% of estimated blood volume is generally considered clinically significant. Excessive bleeding increases the risk of hemodynamic instability, transfusion-related complications, postoperative infection, and prolonged hospitalization. Methods: This narrative review summarizes the current understanding of the incidence, risk factors, anatomical vulnerabilities, and evidence-based strategies for managing IBL in spine surgery through comprehensive literature analysis of recent studies and clinical guidelines. Results: Key risk factors include patient characteristics (anemia, obesity, advanced age, medication use), surgical variables (multilevel instrumentation, revision status, operative time), and pathological conditions (hypervascular tumors, severe deformity). Perioperative medication management is critical, requiring discontinuation of NSAIDs (5–7 days), antiplatelet agents (5–7 days), and NOACs (48–72 h) preoperatively to minimize bleeding risk. The thoracolumbar junction and hypervascular spinal lesions are especially prone to bleeding due to dense vascular anatomy. Evidence-based management strategies include comprehensive preoperative optimization, intraoperative hemostatic techniques, antifibrinolytic agents, topical hemostatic products, cell salvage technology, and structured transfusion protocols. Conclusions: Effective management of massive IBL requires a multimodal approach combining preoperative risk assessment and medication optimization, intraoperative hemostatic strategies including tranexamic acid administration, advanced monitoring techniques, and coordinated transfusion protocols. Particular attention to perioperative management of anticoagulant and antiplatelet medications is essential for bleeding risk mitigation. Understanding patient-specific risk factors, surgical complexity, and anatomical considerations enables surgeons to implement targeted prevention and management strategies, ultimately improving patient outcomes and reducing complications in high-risk spine surgery procedures.

## 1. Introduction

Massive intraoperative blood loss (IBL) during spinal surgery represents a serious and potentially life-threatening complication, particularly in complex procedures such as spinal deformity correction, multilevel fusion, tumor resection, or revision surgeries. Although no universal definition exists, a threshold of more than 1500 mL or 20% of the estimated blood volume is commonly cited in the clinical literature [1,2].

Excessive intraoperative bleeding can lead to profound hemodynamic instability, tissue hypoperfusion, coagulopathy, and transfusion-related complications. It is associated with increased postoperative morbidity, prolonged hospitalization, heightened risk of infection, and the possibility of irreversible neurological deficits. Moreover, the frequent need for allogeneic blood transfusion in such scenarios has been linked to immune modulation and poorer long-term outcomes [3,4,5,6].

Despite significant advances in surgical techniques, perioperative hemostatic strategies, and anesthetic management—including antifibrinolytic therapy, intraoperative cell salvage, and structured patient blood management protocols—massive blood loss continues to pose a major clinical challenge in spine surgery. Among pharmacologic strategies, tranexamic acid (TXA) has emerged as a cornerstone in the prevention of excessive bleeding [7,8,9,10]. TXA, a synthetic lysine analog, acts by reversibly blocking lysine-binding sites on plasminogen, thereby inhibiting fibrinolysis and stabilizing clot formation [11,12,13,14,15]. It has been shown to substantially reduce estimated blood loss (by 400–1000 mL on average) and transfusion requirements (by 30–60%) across a wide spectrum of spinal procedures, including pediatric scoliosis correction, tumor resection, and multilevel instrumentation [7,16,17,18,19,20,21]. Meta-analyses further confirm its favorable safety profile in both adult and pediatric populations, with no significant increase in thromboembolic events when administered appropriately [7,9,10,14,17].

Early identification of high-risk patients, meticulous intraoperative strategies to minimize bleeding, and timely activation of massive transfusion protocols are essential to optimizing perioperative outcomes [15,22,23,24,25,26,27]. This narrative review aims to summarize the current understanding of intraoperative blood loss in spine surgery, focusing on its epidemiology, risk factors, and management strategies, while highlighting areas for future research and clinical improvement.

## 2. Epidemiology of Intraoperative Blood Loss

Global elevation of spine complex surgeries has been matched by a correspondingly growing body of research on anxiety regarding intraoperative blood loss (IBL), particularly in spinal deformity correction, oncologic, and multi-level instrumentation surgeries [1,4,16,28,29]. Though exact epidemiologic data are limited, available literature would indicate that severe blood loss is a feature of 10–40% of spine complex surgeries depending on indication of surgery, surgical technique, and comorbid status of patient [2,8,11,16,17,23,30].

Adult spine deformity corrections and tumor resections are among those processes that have among the highest rates of transfusion and blood loss estimate. As many as one-third of high spine surgeries in North American and European populations have been known to have an EBL of more than 1000 mL, and oncological resections have been known to do so in upwards of 50% of all cases [3,22]. Aging elderly populations that now have spine surgery to many due to spine degenerative changes and risk of fracture have increasingly delicate vascular structures and fewer physiological reserves that make bleeding risk even worse [4]. Similarly, children having corrective spine surgery for scoliosis or congenital deformities are also predisposed to extensive blood loss that is subject to perioperative transfusion [7,11,25,31,32,33].

Trends in operative approach (e.g., anterior vs. lateral vs. posterior approach and minimally invasive spine surgery) and burgeoning pharmacologic intervention such as institution of tranexamic acid (TXA) have further affected the epidemiology of IBL in past years [1,17]. Appreciation of these patterns of epidemiology is critical to optimized allocation of resources, perioperative planning, and protocol development whose aim is to prevent blood loss as well as blood transfusion requirements in spine surgery.

## 3. Incidence and Definition of Massive Intraoperative Blood Loss

The likelihood of experiencing significant intraoperative blood loss varies widely depending on the surgical approach and case complexity. For minimally invasive spine surgeries (MIS), the risk of major bleeding is typically low—under 5%. However, in more complex procedures like deformity corrections or tumor-related operations, that risk can rise above 30% [4,16,25,33,34,35]. In particularly demanding cases, such as en bloc tumor removals or revision surgeries, estimated blood loss may range anywhere from 500 mL to more than 3 L [4,27,36,37].

Several factors influence this variability, including the part of the spine being operated on, the number of spinal segments involved, underlying medical conditions, and whether the patient has highly vascular lesions. Given this complexity, thorough pre-surgical planning—especially identifying risk factors and anticipating the need for blood transfusion—is key to reducing the chance of serious intraoperative issues [11,22].

## 4. Risk Factors

Various patient-specific factors, such as surgery and anatomy, affect the risk and volume of intraoperative blood loss (IBL) during spine surgery. Knowing these risk factors helps in specific prevention methods and preoperative adjustments [4,11,23,38,39,40]. We organized our conceptual framework of risk factors into four categories: patient-related factors, surgery-related factors, anatomical properties, and comorbidity-associated factors (as depicted in Table 1).

### 4.1. Patient-Related Factors

Various patient-related factors have been found to substantially raise the risk of acute IBL. Increased age (>65 years), preoperative anemia (hemoglobin < 12 g/dL), and obese (BMI > 30 kg/m^2^) status have all been linked to increased rates of transfusion. Individual hemoglobin concentrations less than 10 g/dL elevate transfusion risk 2.3-fold and blood loss of more than 1500 mL by 45% in deformity surgery [3,4,41,42,43,44].

Hypervascular tumors in spine tumor surgery lead to a mean EBL of about 1200 mL more than that of hypovascular tumor resections (2300 mL vs. 1100 mL, *p* < 0.001) [22,23,35,39].

### 4.2. Operative Factors

Operative parameters are among the most predictive factors of catastrophic IBL. Three or more levels of instrumentation have much higher rates of blood loss and transfusion. For multilevel lumbar fusion, mean EBL is 1122 mL and transfusion is necessary in close to half of cases [45]. Instrumentation of 10 or more levels has resulted in average EBLs of more than 2100 mL and transfusion rates of more than 60% [1,8,38].

Posterior approaches, particularly in deformity or revision surgery, have a much higher risk of bleeding compared to anterior or minimally invasive surgical (MIS) approaches [46,47]. Anterior approaches in tumor surgery have been found to have 1.6-fold higher EBL in comparison to posterior approaches [22].

Operative time is also a sensitive parameter, as every extra hour of deformity correction is predicted to add an extra 200 mL of blood loss [48]. Revision surgeries also have a 25–35% higher risk of bleeding because of scarring and distortion of normal anatomic planes [1,3].

### 4.3. Relevant Anatomy

The risk and extent of intraoperative blood loss (IBL) during spine surgery significantly depend on spinal anatomy and blood vessel architecture. Some areas and approaches have an inordinate risk because of major vessels’ proximity, epidural venous plexus density, and surrounding structure vascularity.

### 4.4. Thoracolumbar and Lumbosacral Regions

Thoracolumbar junction (T10–L2) is especially predisposed to severe bleeding in posterior or lateral exposures. This transitional zone is endowed with an elaborate epidural venous plexus that may become engorged under accompanying intra-abdominal pressure or prone positioning. This plexus injury is liable to cause diffuse, challenging bleeding control most especially in deformity and tumor surgery [3,8,45].

The transition area between the fixed thoracic spine and the mobile lumbar spine necessitates wide dissections of many anterolateral musculature multilevel fusions and deformity corrections. This is an area replete with highly developed epidural venous plexus that is devoid of venous valves and is subject to engorgement and hemorrhage when prone positioning is employed [1,8].

Lumbar spine (L2–S1) is also at a high risk of bleeding because of lumbar segmental arteries that arise from the aorta of abdomen and descend through the vertebral bodies and neural foramina. During anterior lumbar interbody fusion (ALIF) below L4–L5 and L5–S1 slipping of the iliolumbar artery, mid sacral artery, or common iliac vessels may lead to disastrous hemorrhage. Preoperative vascular imaging is usually indicated in high-risk situations to visualize anatomical variations [3].

### 4.5. Cervical Spine

Generally, cervical spine surgeries have less blood loss than thoracolumbar surgery. Anterior cervical methods have a risk of blurring injury to the vertebral artery, especially along C5–C6 and C6–C7 levels where the array migrates from the transverse foramen. While an exceedingly rare (<1%), vertical array injury can precipitate unexpected hemorrhage as well as posterior circulation ischemia [49].

## 5. Pathophysiology

### 5.1. Venous Bleeding: Epidural Venous Plexus

The epidural venous plexus is the most common source of bleeding during posterior spinal surgeries. This valveless, low-pressure network is especially prominent in the thoracolumbar region. Its volume can increase with elevated intra-abdominal pressure, which makes obese patients or those placed in the prone position more prone to venous engorgement. Injuries during dissection, decompression, or pedicle screw placement can result in slow but continuous bleeding, often soaking through sponges and overwhelming suction systems [1,8].

### 5.2. Arterial Bleeding: Segmental and Iliac Vessels

Though less frequent than venous bleeding, arterial bleeding poses a greater danger due to its sudden onset and potential for rapid hemodynamic deterioration. Damage to segmental arteries, the iliolumbar artery, or branches of the common iliac vessels is most commonly encountered during anterior lumbar approaches, especially ALIF at the L4–S1 levels. These injuries can cause pulsatile bleeding and immediate instability, often requiring urgent vascular intervention [3].

In cervical spine surgeries, injury to the vertebral artery—though rare (<1%)—can be devastating. These injuries typically occur during drilling or hardware placement at levels C5–C7, and may lead to severe bleeding and posterior circulation stroke [49].

### 5.3. Tumor and Vascular Lesion Surgery

Hypervascular spinal tumors carry a particularly high risk of substantial intraoperative blood loss. Primary tumors like hemangioblastomas, giant cell tumors, and plasmacytomas, along with metastases from cancers such as renal cell carcinoma, hepatocellular carcinoma, thyroid cancer, and melanoma, are highly vascular. These tumors often develop collateral blood supply from surrounding vessels, making bleeding widespread and difficult to control [22,30,38].

### 5.4. Tumor Vascularity and Bone Bleeding

Spinal tumors with rich vascular networks—such as those from renal or thyroid cancer—pose a significant bleeding risk due to fragile, newly formed vessels. Diffuse bleeding is common during intralesional resections. The median estimated blood loss (EBL) in these oncologic spine procedures often exceeds 1700 mL, particularly when anterior approaches or highly vascular tumors are involved [22,23,30,38,39].

Additional blood loss can also result from bleeding surfaces of exposed cancellous bone during procedures like osteotomies, facetectomies, or corpectomies. This is especially relevant in long-segment fusions or deformity corrections, where prolonged exposure can cause venous congestion and continuous oozing [1,22].

## 6. Intraoperative Recognition and Timing of Bleeding Events

### 6.1. Intraoperative Bleeding Is Typically Phase-Specific

Intraoperative bleeding in spine surgery is frequently phase-dependent and staged by phase of operation. As the operation enters its phase of initial exposure and dissection, bleeding is most frequently defined by venous oozing from the paraspinal musculature and epidural venous plexus. As successive stages of operation reach osteotomy and decompression, bleeding is most frequently from cancellous surfaces of bone and deeper portions of venous plexus. Tumor resections pose a particularly difficult situation because tumor resections are so highly vascularized that resections of tumor tissue are frequently associated with extensive hemorrhage and hemorrhage from adjacent bone. As the operation progresses to its later phase of placing implants and closing the operation, bleeding is most frequently trivial; however, it may tip ongoing losses if bleeding totals have been poorly controlled in a prior phase of operation. Interestingly, as much as 40% of overall intraoperative blood loss in spine deformity operation may accumulate in its phase of correcting or releasing deformity of bone, underscoring significance of this anticipated bleeding as control of bleeding is attempted through all stages of operation [48,50,51,52].

### 6.2. Intraoperative Recognition of Hemorrhage

Major bleeding during spine surgery can present in two distinct ways: suddenly or gradually. Arterial injuries usually cause a rapid and dramatic onset of symptoms, including a sharp drop in blood pressure, bright red pulsatile bleeding, and suction canisters filling quickly with blood. On the other hand, bleeding from veins or cancellous bone tends to develop more slowly. This type may not be noticed right away, gradually obscuring the surgical field and accumulating until significant blood loss has already occurred.

Detecting hemorrhage effectively requires constant, coordinated attention from both the surgical and anesthesia teams [3,37,38,53,54]. The anesthesiologist plays a vital role by tracking hemoglobin levels, blood pressure trends, and fluid balance. Meanwhile, the surgeon must stay alert to subtle indicators during the operation. These include worsening visibility in the surgical site, continuous bleeding from epidural tissues or exposed bone, increasing blood levels in suction or cell salvage devices, and changes in tissue color that may suggest diffuse oozing.

### 6.3. Pathophysiology of Hemorrhage and Coagulopathy

Physiological effects of massive hemorrhage go beyond mere loss of volume. With ongoing bleeding, dilutional coagulopathy from excessive fluid resuscitation and loss of coagulation factors may ensue. At the same time, hypothermia from undue exposure and acidosis from inadequacy of tissue perfusion may emerge. These three disorders—coagulopathy, hypothermia, and acidosis—constitute so-called “lethal triad” of self-propagating disorders that seriously compromise hemostasis and make resuscitation increasingly difficult. Unless promptly identified and corrected, this triad itself may advance to disseminated intravascular coagulation (DIC) and lead to multi-organ failure. Aggressive identification and curing of each of its constituents are thus of paramount importance to saving patient life from potential intraoperative disasters [31,32,33].

### 6.4. Impact of Anticoagulant and Antiplatelet Medications on Bleeding Risk

Current meta-analyses support individualized timing for Direct Oral Anticoagulant (DOAC) interruption based on renal function, drug half-life, and the bleeding risk associated with the planned procedure [55,56]. In patients with normal renal function undergoing elective procedures with low bleeding risk, DOACs should be discontinued 24 to 48 h before surgery. For patients with renal impairment, particularly those receiving dabigatran, this interval should be extended to at least 48 h [55,57]. For high-bleeding-risk procedures, a 48 to 72-h interruption is typically recommended, with longer intervals in patients with significantly reduced creatinine clearance [58,59,60,61,62]. In emergency surgical settings, the presence of residual anticoagulant activity must be assumed, and preparations should include appropriate reversal strategies [55,63].

Systematic reviews consistently demonstrate that routine bridging with heparin increases the risk of bleeding without offering a corresponding reduction in thromboembolic events in most patients on DOAC therapy [56,64]. Therefore, routine bridging should be avoided, as meta-analyses show no overall clinical benefit and a heightened risk of major bleeding complications [56,64]. Bridging anticoagulation may be considered in selected high-risk patients, such as those with mechanical heart valves, a history of venous thromboembolism or stroke within the past three months, or other situations where thromboembolic risk is deemed exceptionally high after multidisciplinary evaluation [56,65]. For patients undergoing cardiac implantable electronic device procedures, uninterrupted anticoagulation has demonstrated superior safety and efficacy compared to interruption with bridging [56].

In patients undergoing transcatheter aortic valve replacement (TAVR), pooled analyses suggest that uninterrupted DOAC therapy can be safely continued in selected individuals. This strategy maintains comparable bleeding rates while reducing stroke risk compared to interruption protocols [55]. Similarly, patients undergoing cardiac device implantation benefit from maintaining anticoagulation, which lowers the incidence of thrombotic complications without increasing the risk of major bleeding [56]. In orthopedic procedures such as hip and knee arthroplasty, DOACs provide effective prophylaxis against venous thromboembolism and are comparable in safety to low molecular weight heparin [66]. For cancer surgeries, extended-duration prophylaxis with DOACs effectively reduces postoperative VTE risk while maintaining an acceptable bleeding profile [67,68].

The use of perioperative antiplatelet therapy requires careful assessment. Dual antiplatelet therapy is associated with a significant increase in bleeding risk and should be managed on an individual basis. Aspirin monotherapy can often be safely continued for many elective procedures [69,70,71]. However, the concomitant use of aspirin and DOACs necessitates a thorough bleeding risk evaluation [34,39,69,70,71,72,73].

Perioperative anticoagulant and antiplatelet medicine management is an important bleeding risk reduction practice of spine surgery. These drugs profoundly affect hemostatic function and deserve close consideration to weigh bleeding against thrombotic risk. NSAIDs and both selective and non-selective COX inhibitors have an inhibitory effect on platelet aggregation by inhibiting synthesis of thromboxane A2 [34,39,72,73].

Even low-dose aspirin (75–100 mg/day) will raise bleeding risk by 30–50% during spine surgery. Aspirin has an irreversible antiplatelet effect whose duration is equal to that of a platelet lifespan (7–10 days) and requires timely discontinuous preoperative stops. Preoperatively, other NSAIDs such as ibuprofen, naproxen, and diclofenac have reversible antiplatelet activity and must be stopped 5–7 days before to permit complete drug clearance and restoration of platelet functions.

Beyond amplified intraoperative blood loss alone, clinical relevance exists in NSAID-induced bleeding. Patients persevering in NSAIDs during spine surgery have higher epidural hematoma formation rates, higher wound healing difficulties, and reoperation requirements. By virtue of their anti-inflammatory action, NSAIDs can negatively affect an initial inflammatory reaction required in hemostasis [34,53,74,75].

P2Y12 inhibitors (clopidogrel, prasugrel, ticagrelor) have a high risk of bleeding in spine surgery [39]. Perioperative bleeding is doubled by clopidogrel 40–60% and must be stopped 5–7 days before operation to give time to have good turnover of platelets. Prasugrel and ticagrelor have comparable bleeding risk but distinct pharmacokinetic profiles giving different timing of discontinuation [35,74,75]. For unstable patients or those with high cardiovascular risk in the past, a decision to withdraw antiplatelet therapy is made through multidisciplinary consultation involving cardiology to weigh thrombotic against bleeding risk. Timing of stent insertion, stent type (bare metal versus drug eluting), and predisposing cardiovascular risk factors all affect this decision-making process.

Direct Oral Anticoagulants (DOACs) are becoming increasingly employed clinically and pose characteristic perioperative challenges in spine surgery because of their pharmacologic profiles and bleeding risk envelopes [34,35,39,72]. As compared to warfarin, NOACs—also referred to as novel oral anticoagulants—share a distinctive rapid onset and offset of action. Due to this pharmacokinetic predictability, perioperative management is more standardized but simultaneously requires much more exact timing of discontinuation and possible reversal. Among the NOACs, dabigatran is a direct thrombin inhibitor that has the longest class half-life and high renal clearance. As such, its discontinuation needs to be appropriately individualized to renal function. For patients with normal renal function, dabigatran should be discontinued 48 to 96 h prior to surgery. For patients with decreased renal function—most particularly when creatinine clearance is less than 50 mL/min—discontinued use should be as long as five days to allow a small risk of residual anticoagulant effect to wane prior to surgery [34,35].

Factor Xa inhibitors, including rivaroxaban, apixaban, and edoxaban, usually necessitate a 48- to 72-h discontinuation time before high-bleeding-procedure like spinal surgery [34,39,55,56,72,73,76,77,78,79]. Routine coagulation assays are not reliable for monitoring DOAC activity. Instead, drug-specific assays should be used when available, and hematology consultation is recommended for interpretation. For patients undergoing cancer-related surgery, extended thromboprophylaxis with DOACs offers a convenient oral alternative to subcutaneous low molecular weight heparin [67,68]. Apixaban, even having a short half-life among the Factor Xa inhibitors, is given twice a day and consequently necessitates an equal preoperative interruption interval to completely eliminate drug and lower perioperative bleeding risk. Reversal of NOACs is possible but remains difficult and resource-based. In the event of urgent surgery or major bleeding, targeted DOAC reversal agents should be used when available [63]. Special reversal agents have been made: idarucizumab is exceedingly efficient in reversing dabigatran’s anticoagulant effect, whereas andexanet alfa has been proved to reverse Factor Xa inhibitors’ effects. These agents, however, carry a price tag and might not be universally present in clinical situations. When special reversal agents are not present or are against medical indications, prothrombin complex concentrates (PCCs) could be employed as a replacement to quickly reverse anticoagulant effects [29,35,77,80,81,82,83,84]. Idarucizumab is an effective and specific reversal agent for dabigatran and is indicated in emergency scenarios [63]. Institutions should develop formal protocols for DOAC reversal, including well-defined activation criteria and multidisciplinary team involvement to ensure timely and appropriate care [63]. Standardized protocols should be implemented for high-risk procedures, with open communication among surgical, anesthetic, and medical teams to ensure safe and effective anticoagulation management [55,56,85,86,87].

### 6.5. Clinical Implications and Risk Stratification

Several studies have indicated that spine surgery performed in continuation of anticoagulant or antiplatelet therapy results in a two- to threefold incidence of major bleeding complications such as epidural hematomas, wound problems, and reoperation [39,72]. This complication is most significant in some high-complexity spine surgery [24,25,76,85,87]. Those spine surgeries that have multi-instrumented levels have higher disruption of blood vessels and longer operative duration and thus have a higher risk of bleeding. Revision surgeries, especially those that have extensive scar formation, have difficulties in tissue dissection and hemostatic control. Anterior cervical spine surgery is of particular risk because it is close to principal blood vessels including the carotid and vertebral arteries. Tumor resections that are particularly those resections of hypervascular tumors have a marked risk of hemorrhage during and after surgery [30,77].

Even with these perilous situations, it is just as dangerous to abruptly discontinue antithrombotic agents in highly thromboembolic risk patients. Patients who have received a recent stent in a coronary artery—particularly those receiving drug-eluting stents within six months of stent placement—are highly susceptible to in-stent thrombosis if their antiplatelet regimen is discontinued. Patients with atrial fibrillation and high CHA_2_DS_2_-VASc scores are also at high risk of a stroke if anticoagulation is discontinued. This is also true for those having a documented history of ischemic stroke or transient ischemia attack, mechanical heart valves, or a recent occurrence of venous thromboembolism. These types of patients should have their anticoagulation discontinued on an extreme caution scale [35,53,74,75].

In some high-risk situations, bridging techniques can be used to lessen thromboembolic risk. This is usually done through perioperative short-acting agents like low molecular weight heparin or unfractionated heparin. Bridging therapy itself is itself highly associated with perioperative bleeding risk and must only be used when thromboembolism risk outweighs bleeding risk by a clear margin. This requires a subtlety-calibrated risk-versus-benefit analysis that balances a number of important variables such as the patient’s specific thrombotic risk category, perioperative bleeding risk estimate, presence of rapid reversal agents, and capacity of an institution to have intense postsurgical monitoring and complication control [59,63,64,65,66,67,68].

Postoperative resumption of antithrombotic therapy also needs to be thoughtfully planned and individualized to the clinical status of each patient. Timing is most dependent upon the thoroughness of hemostasis obtained and delayed bleeding risk. As a general rule, antiplatelet agents can usually be resumed within 24 to 48 h of surgery if hemostasis is solid and no clinical evidence of ongoing bleeding exists. Anticoagulant therapies frequently necessitate a longer wait—usually 48 to 72 h or longer—depending upon surgical procedure complexity and individual patient bleeding risk [57,58,59,63,64]. For high-risk patients, a staged approach to resumption of antithrombotic agents may be useful, starting short-acting agents first and later reversing to long-term anticoagulation [59,63,64,88].

During the perioperative phase, careful clinical observation is necessary to reveal predilection signs of bleeding or thrombotic adverse events. Algorithms must be established to direct practitioners to treat such events expeditiously and efficiently to allow safe conduct of this most vulnerable transition phase.

## 7. Management Strategies

Effective bleeding control in spine surgery relies on a multifaceted approach. Success hinges not just on surgical technique and resuscitative strategies, but also on careful preoperative pharmacologic planning—particularly around medications that affect clotting. These drugs influence systemic coagulation pathways and, when combined with the spine’s rich vascular supply and close proximity to vital neural structures, demand meticulous perioperative oversight to maintain both safety and surgical efficiency [55,56,59,63,78,79].

Early detection and proactive management of bleeding during surgery are essential to prevent serious complications. Mechanical hemostatic techniques serve as the primary defense and should be applied consistently. These include direct pressure on bleeding areas and the use of bipolar electrocautery, which allows for targeted coagulation while minimizing heat damage to nearby tissues. In addition to mechanical methods, topical agents play a key supporting role. Common options include gelatin sponges and oxidized cellulose, which help form a scaffold for clotting, as well as fibrin sealants and bone wax—particularly useful for bleeding from exposed cancellous bone during procedures like osteotomies or corpectomies [1,8,80,81,89,90,91]

Systemic antifibrinolytic therapy also plays a vital role, especially tranexamic acid (TXA). TXA works by blocking plasminogen activation, which stabilizes clot formation. A growing body of randomized controlled trials and meta-analyses supports its effectiveness—demonstrating reductions in estimated blood loss by as much as 800 mL and decreased transfusion requirements by 30–50%, without an associated rise in thromboembolic events [8,11,18,23,30,33,51,52,53,69,70,71,74,92,93].

**Figure 1:**
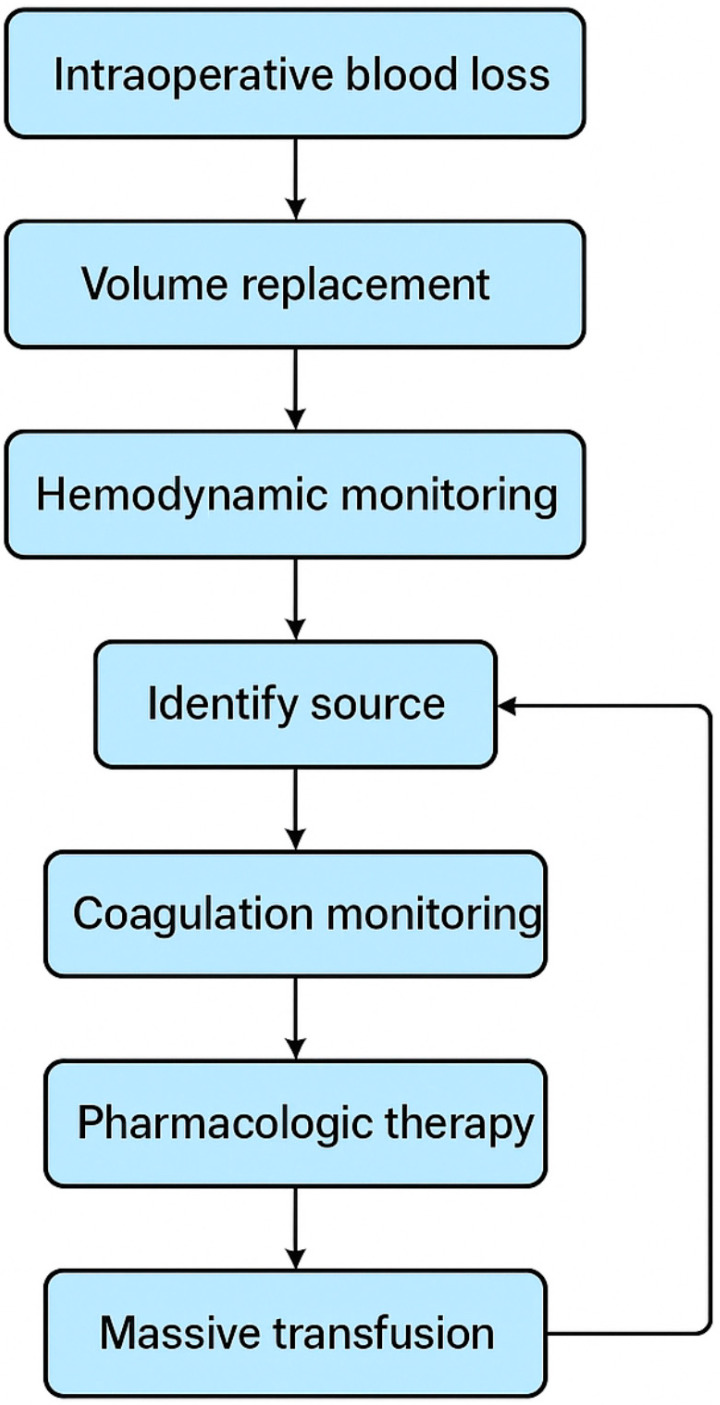
Management of massive intraoperative blood loss in spine surgery.

Figure 1 illustrates the stepwise approach to managing massive intraoperative blood loss in spine surgery.

Several recent systematic reviews and network meta-analyses are reported to say TXA decreases intraoperative, postoperative and overall blood loss and oftentimes decreases transfusion volume in spine surgeries on adults and children; magnitude dependent upon procedure level and dose [16,18,22,33,45,48,49,69,72,80,94,95]. A network meta-analysis conducted on the incidence among children found a relative decrease in bleeding around the perioperative period (0.71 ratio of means, 95% CI 0.62–0.81, *p* < 0.001) with the use of TXA in spine surgery [19,70,71,96]. Network meta-analyses conducted on large or multilevel corrective procedures also indicate decreased intraoperative and overall blood loss and decreased distribution of blood for transfusions when the TXA is given intravenously [5,7,9,10,13,96]. Latest dose-centric network and meta-analyses, along with pooled studies, have compared low-dose and high-dose regimens while evaluating optimal intravenous strategies. Higher-dose regimens are generally associated with greater reductions in blood loss, although there is limited conclusive evidence supporting their superiority in terms of safety. Frequentist network meta-analyses conducted in adolescent idiopathic scoliosis, along with intravenous dosing analyses, examined a range of regimens and concluded that higher-dose protocols, typically involving a bolus followed by infusion, lead to significantly lower blood loss and transfusion requirements compared to single or low-dose approaches [12,15]. A meta-analysis specifically comparing high- versus low-dose protocols in adolescents demonstrated that high-dose regimens produced more substantial reductions in blood loss in pooled data sets [14]. Several pooled analyses have demonstrated that high-dose regimens, particularly those combining a bolus with a continuous infusion, are associated with greater reductions in measured blood loss compared to low-dose bolus or placebo in the setting of complex spinal deformity surgeries [14,15]. Network meta-analyses further suggest that a bolus followed by infusion is the most effective intravenous approach for prolonged surgical procedures, although specific mg/kg dosing targets vary depending on the study and patient population [12,15]. It is important to note that high-dose protocols necessitate careful monitoring due to the potential for rare adverse events, emphasizing the need for individualized risk assessment [10]. Comparative analyses indicate that intravenous, oral, and topical routes of tranexamic acid (TXA) administration are each effective in reducing perioperative blood loss. Randomized controlled trials have shown that oral TXA demonstrates efficacy comparable to intravenous administration, while the combined use of topical and intravenous TXA may offer additional benefits in specific surgical contexts. Meta-analyses of randomized trials report no significant differences in intraoperative or total blood loss between oral and intravenous administration. However, pooled data demonstrate a statistically significant reduction in postoperative drainage volume with oral TXA [19]. Systematic reviews comparing topical and intravenous routes yield mixed findings. Intravenous TXA tends to provide more consistent reductions in both visible and hidden blood loss, whereas topical application may reduce local wound drainage and offers an alternative when systemic exposure is a concern [9,97]. A recent pooled analysis found that combining intravenous and topical TXA reduced postoperative blood loss more effectively than either route alone in several studies involving spinal deformity fusion procedures [77,98,99]. Intravenous administration is supported by a robust body of evidence and remains the most reliable approach for major multilevel and spinal deformity surgeries [9,13]. Oral TXA has been found non-inferior to intravenous TXA in many thoracolumbar fusion trials and is considered both cost-effective and convenient for elective procedures [19,100,101]. Topical TXA may serve as a useful adjunct to reduce local drainage, although current evidence is inconsistent regarding its effectiveness in reducing total blood loss compared to intravenous administration alone [97,102]. The combination of intravenous and topical TXA may provide additive benefits in reducing postoperative blood loss, particularly in selected deformity cases [76]. Safety, pediatric findings, blood loss outcomes, and recommendations.

Safety data drawn from meta-analyses are generally reassuring regarding the routine use of TXA in spine surgery. However, the variability in study reporting and the statistical fragility of some trials warrant a cautious interpretation of results. In pediatric populations, pooled analyses have demonstrated a clear benefit in reducing perioperative bleeding and transfusion requirements, without consistent evidence of an increased risk for thromboembolic complications [103]. Larger, procedure-specific reviews confirm reductions in intraoperative and total blood loss, as well as lower transfusion volumes, without a corresponding increase in overall complication rates or thromboembolic events. Nevertheless, signals from non-spine surgical settings and methodological limitations in certain RCTs support a conservative and individualized approach to TXA administration [5,7,10,102].

Systematic reviews in spinal surgery have not shown a consistent increase in venous thromboembolism associated with perioperative TXA use, though event rates remain low and findings are heterogeneous. Evidence specific to oncologic spine surgery is limited and should be assessed on a case-by-case basis [7,10]. High-dose TXA has been associated with seizures in cardiac surgery literature, and this concern has also been raised in dose-ranging trials. Clinicians should carefully weigh the potential benefits against the risk of neurotoxicity, particularly in patients with preexisting neurologic conditions or vulnerability [10]. In pediatric spine surgery, TXA is widely used and considered effective, especially in adolescent scoliosis protocols. Dosing in children should follow regimens supported by clinical trials, including bolus with or without infusion, and adhere to institutional pediatric guidelines, as pharmacokinetic profiles vary with age [14,102,103]. Across pooled analyses, TXA has been shown to significantly reduce intraoperative, hidden, and total blood loss, as well as transfusion requirements in multilevel, deformity, and instrumented lumbar spine procedures [5,13,102].

In the case of major hemorrhage, activation of a Massive Transfusion Protocol (MTP) is warranted. An MTP typically employs a 1:1:1 ratio of packed red blood cells (PRBCs), fresh frozen plasma (FFP), and platelets, guided by viscoelastic testing (e.g., TEG, ROTEM) or standard coagulation assays (PT/INR, aPTT, fibrinogen levels). Correction of hypocalcemia, hypothermia, and acidosis is critical during resuscitation [80,81,82,83].

### 7.1. Preoperative Assessment and Optimization

Perioperative management of antithrombotic agents, including non-steroidal inflammatory agents (NSAIDs), antiplatelet drugs, and new oral anticoagulants (NOACs), is an important part of prevention of surgical bleeding risk, especially in high-blood-loss surgeries like spine surgery. All of these agents of a class affect hemostasis through different mechanisms of action and warrant personalized timing of their administration according to pharmacokinetics, renal function, and thrombotic risk of a patient [11,16,17,22,35,53,74,75,80].

NSAIDs, which are common treatments for chronic musculoskeletal pain, have their action mainly as a reversibly inhibitory effect on cyclooxygenase-1 (COX-1) to lower thromboxane A2 production and disrupt platelet aggregation. While this action is reversible and generally short-acting, its clinical impact is magnified in situations of high vascular exposure in surgery or when co-administered with additional anticoagulants. Present recommendations would be to discontinue the use of non-selective NSAIDs 5 to 7 days before surgery to permit adequate clearance and restoration of normal platelet activity, although this interval might depend on that particular drug’s half-life. By way of illustration, ibuprofen might only require a 24-h washout in normal subjects, while longer-acting agents like piroxicam would require a longer washout. New evidence now indicates that selective inhibitors of COX-2 agents like celecoxib might have safer perioperative usage in some situations because of their near-negligible effects on coagulation of the blood and yet belong to a class of drugs that have been found to have cardiovascular adverse effects [47]. It is indeed of particular importance to appreciate that bleeding risk from NSAID usage significantly augments when these agents are co-administered with anticoagulants to such an extent that studies conducted recently from a population-based arena have indicated an over twofold incidence of major hemorrhagic manifestations like gastrointestinal bleeding as well as intracranial bleeding [29,35,77,80,81,82,83,84].

Aspirin, even at low daily dosage of 75–100 mg, irreversibly inhibits platelet COX-1 and consequently provides a sustained antiplatelet effect that lasts as long as the affected platelets reside in circulation—about 7 to 10 days. Therefore, aspirin is usually withheld for 7 to 10 days before elective surgery to minimize bleeding complications. Nevertheless, this consideration must be balanced against thrombotic events risk in patients with a recent coronary stent or past acute coronary syndromes. Meta-analyses have indicated that continuation of low-dose aspirin during the perioperative phase modestly enlarges major bleeding risk (relative risk ~1.31), yet simultaneously lowers thromboembolic complication risk (relative risk ~0.74) [35,53,74,75,104]. Maintenance of aspirin therapy through the perioperative phase in select patients having a high cardiovascular risk may be warranted and must be determined by multidisciplinary consensus of cardiology, anesthesia, and surgery teams.

Perioperative management of P2Y12 inhibitors—specifically clopidogrel, prasugrel, and ticagrelor—offers an even bigger challenge given their strong and frequently irreversible inhibition of ADP-mediated platelet aggregation. These agents have been linked to a 40 to 60 percent risk increase in perioperative bleeding risk, especially in spinal, vascular, and cardiac surgical situations. As such, existing recommendations suggest discontinuation of clopidogrel and ticagrelor at least five days before operation and prasugrel for at least seven days due to its longer duration of action as well as higher potency [41,73,74,75,104]. Note that bridging regimens employing low molecular weight heparin or unfractionated heparin during perioperative P2Y12 inhibitor discontinuance are typically ineffective in terms of inhibiting platelet-proximate thrombotic events like stent thrombosis and indeed might increase bleeding risk without conferring protective benefit. As such, in patients having received recent percutaneous coronary intervention or having a complex cardiac history, perioperative antiplatelet management must be decided upon through multimodal risk stratification on a collaborative basis and preferably with input from treating cardiologist.

Novel oral anticoagulants (NOACs), or direct oral anticoagulants (DOACs), like dabigatran, rivaroxaban, apixaban, and edoxaban, have been widely employed for thromboembolic prophylaxis and treatment because of their predictable pharmacokinetics and avoidance of regular monitoring. Nevertheless, their short half-life and excretion through the kidneys require timely discontinuation before operation. Generally, NOACs must be discontinued 48 to 72 h before high-bleeding-risk operations like multilevel spinal fusion, with longer discontinuation periods (up to 96 h or longer) necessary for dabigatran in patients with poor renal function [41,105,106]. The historic PAUSE trial proved that a standardized strategy of DOAC interruption without heparin bridging was safe and efficient in minimizing bleeding difficulties without thwarting thromboembolic events in the perioperative setting. Bridging routine utilization of parenteral anticoagulants is thus discouraged as this merely increases bleeding risk without important thrombotic protection. Special reversal agents are now commercially available—idarucizumab against dabigatran and andexanet alfa against factor Xa inhibitors—though their considerable cost and restricted availability make their utilization judicious only in emergent situations. After operation, DOACs are resumed 24 h following low-bleeding-risk intervention and 48 to 72 h afterwards following high-bleeding-risk operation, depending on verification of hemostasis and no bleeding from a far-removed operation site [29,35,37,41,42,59,63,64,65,66,67,68,80,81,82,83,84,105,106].

Perianesthetic discontinuation of NSAIDs, antiplatelets, and NOACs must be directed by the pharmacologic properties of each drug, the bleeding risk of the intended procedure, and the patient’s own thrombotic risk. These management techniques must be tailored and high-risk recommendations must be made in consultation with multidisciplinary teams to allow for the safest of anesthetic results.

Preoperative laboratory optimization is an important role that is used to reduce perioperative bleeding risk as well as have physiological reserves that are adequate during surgery. Optimal hemoglobin levels should be above 12 g/dL whenever possible through supplementation of iron or by treating those hidden causes of deficiency such as nutritional deficiency or chronic disease. Coagulation parameters such as prothrombin time (PT), international normalized ratio (INR), activated partial thromboplastin time (aPTT), and platelet count should be adequately assessed and corrected before surgery to have normal hemostatic function. Preoperative autologous blood donation can be a consideration as a method to attenuate allogeneic transfusions in selected elective surgeries that have a bleeding risk that is high. Comprehensive nutritional assessment should also be done in such a way that it leads to focused intervention to have optimal protein reserves as well as to correct deficiency of important vitamins especially those that are part of coagulation pathway such as vitamin K, vitamin B12, and folate [34,35].

### 7.2. Intraoperative Hemostatic Strategies

Pharmacologic approaches remain an important part of intraoperative hemostasis when high blood loss is predicted during procedures. Of antifibrinolytic agents, tranexamic acid (TXA) is commonly employed because of its effectiveness and safe character. It is given as an intravenous loading dose of 10–15 mg/kg and an ongoing infusion of 1–2 mg/kg/h as required during procedure [7,9,10,20,21,107]. Various studies have confirmed that intravenous and topical application of TXA administered through two presents a superior blood-sparing effect compared to application through either of these alone. When TXA is contraindicated as is true in active thromboembolic disease past or, relatively, seizure history—aminocaproic acid is a replacement antifibrinolytic agent that can be used. No matter which agent is employed, close intraoperative and postsurgical monitoring is necessary to look for signs of thrombotic problem so that appropriate action could be made to protect patient [23,37,40,42,43,44,83]. Intravenous tranexamic acid (TXA) should be routinely utilized for multilevel spinal fusion and deformity procedures. In elective thoracolumbar fusion cases without contraindications to topical agents, oral TXA represents an evidence-based and cost-effective alternative [13,19]. In patients undergoing high-risk deformity correction, the combined use of topical and intravenous TXA may offer incremental benefits in minimizing postoperative blood loss [76]. To optimize clinical practice, there is a clear need for large-scale, pragmatic randomized controlled trials and prospectively maintained safety registries. These studies should be stratified by dosing regimen, route of administration, oncologic status, and pediatric age to better define optimal protocols, assess the frequency of rare adverse events, and quantify absolute reductions in transfusion requirements [10,12].

Topical hemostatic agents are useful adjuncts to systemic control and surgical technique. Fibrin sealants and thrombin-based products are especially useful in controlling localized bleeding. Oxidized cellulose and gelatin-based matrices are generally used to control diffuse oozing due to their absorptive and thrombogenic functions. Control of bleeding from cancellous bone surfaces can be achieved using bone wax, although this application must be discreet due to its potential to cause a foreign body reaction and hinder bone healing. Flowable hemostatic agents can be a customized solution to achieving hemostasis when dealing with irregular or difficult anatomical surfaces [80,82,83,108].

Mechanical hemostasis is just as important and starts with thorough surgical technique. This consists of gentle tissue handling and maintaining anatomical plane to keep to a minimum unnecessary vascular damage. Bipolar electrocautery is the preferred choice for selective vessel closing, as it provides site-specific coagulation and limited collateral thermo injury. Selective controlled hypotension to a mean systemic pressure of 65 to 70 mmHg may be used in select patients where it is not contraindicated to limit intraoperative blood loss. Proper patient positioning is also important as it can effectively limit venous congestion in this field of operation. Utilization of adjunctive technologies, including ultrasonic dissection instruments, complements blood conservation by adding to soft tissue dissection efficiency and selectivity [16,22,80,82,108].

### 7.3. Blood Conservation and Transfusion Management

Massive transfusion in the context of spine surgery is defined as intraoperative or perioperative blood loss exceeding 2500 mL or the requirement for more than four units of packed red blood cells within a 24-h period [109]. Preoperative indications include planned long-segment spinal fusions involving more than four vertebral levels, three-column osteotomies, and surgeries for vascular spinal tumors [110]. Intraoperative activation of a massive transfusion protocol (MTP) is warranted in situations of rapid and ongoing hemorrhage that result in hemodynamic instability or when anticipated total blood loss is expected to surpass established thresholds [109,111].

Intraoperative autologous blood salvage is an established method of reducing exposure to allogeneic blood products and is especially useful in spine surgery where blood loss is anticipated to be more than 1000 mL [35,92,112,113,114]. This technique entails the collection and filtered reinfusion of a patient’s own blood once it is shed to preserve a patient’s red cell mass without incurring immunologic and infectious transmission from blood transfusion. Various limitations must be put into consideration to enable a patient to undergo this procedure safely including malignancy and active infection presence and intraoperative use of agents that will soil blood once salvaged. Patient protection requires strict control of quality once collection and reinfusion of blood is to be done including proper anticoagulation, mechanical filtration and close monitoring for signs of hemolysis or contamination.

The perioperative transfusion approach should be restrictive in nature and is spurred by strong evidence in all of surgery specialties. For hemodynamically stable patients without cardiovascular disease, red blood cell transfusion is generally only advocated when hemoglobin levels reach below 7 to 8 g/dL. Conversely, in patients with severe cardiovascular comorbid illness, higher thresholds of 8 to 9 g/dL could be optimal to preserve appropriate oxygen delivery and prevent compilation of ischemia. Platelets are required in patients having active bleeding when the count of platelets is less than 50,000/μL, or in those having a count of less than 100,000/μL when subject to procedure having high risk of bleeding. Fresh frozen plasma is required to correct coagulopathy especially when the patient has an international normalized ratio (INR) of more than 1.5 concurring in active hemorrhage.

In a situation of significant intraoperative blood loss—delimited as more than 1500 mL, more than 20% of an estimate of a patient’s blood volume, or with clinical signs of hemorrhagic shock—a Massive Transfusion Protocol (MTP) should be activated [34]. MTP allows rapid balanced resuscitation through immediate administration of packed red blood cells, fresh frozen plasma, and platelets in a 1:1:1 proportion and prevents dilutional coagulopathy as a result, facilitating hemodynamic stability. Early and simultaneous activation of hematology, blood bank support services, and critical care teams is important to facilitate efficient utilization of the protocol. Constant monitoring is necessary in this regard and serial laboratory analyses including complete blood count (CBC) are done along with coagulation studies (PT, aPTT, INR) to monitor fibrinogen levels and arterial blood gas to direct transfusion as well as resuscitative efforts. Effective implementation of an MTP requires immediate availability of blood products in a 1:1:1 ratio of red blood cells, fresh frozen plasma, and platelets. Routine use of TXA, as guided by institutional patient blood management (PBM) protocols, has been shown to significantly reduce blood loss and transfusion requirements [20,21]. Critical adjuncts include autotransfusion strategies such as acute normovolemic hemodilution (ANH) and intraoperative cell salvage. The use of topical hemostatic agents further supports intraoperative hemostasis [95,103]. Clear role assignments, activation checklists, and robust communication with the blood bank are essential for protocol efficiency and safety [78]. Comprehensive risk stratification should be conducted during the surgical planning phase to identify patients at high risk of significant blood loss. This includes those undergoing long-segment fusions, three-column osteotomies, or surgery for neoplastic spinal conditions [79,110]. Correction of preoperative anemia using intravenous iron or erythropoietin, based on local protocols, is mandatory [110]. High-risk cases should be reviewed in multidisciplinary conferences involving the surgical team, anesthesiologist, and transfusion medicine specialists [78].

Intraoperative strategies must focus on minimizing blood loss through strict blood pressure control and protocol-driven use of TXA, in addition to ANH and cell salvage where major hemorrhage is anticipated [20,21,107]. The application of active hemostatic matrices has been shown to significantly reduce blood loss, particularly in spinal deformity procedures [115]. Hemoglobin levels, platelet counts, and coagulation profiles should be monitored in real-time, while limiting routine postoperative lab draws to clinically indicated scenarios [116]. Institutions should maintain a quality control registry to document MTP activations, product usage, and related complications, with regular audits and updates to the protocol [78].

### 7.4. Advanced Hemostatic Monitoring

Viscoelastic testing modalities, including thromboelastography (TEG) and rotational thromboelastometry (ROTEM), provide real-time, dynamic measurements of the complete coagulation process from initiation of clot through to fibrinolysis. These modalities enable identification of select hemostatic deficits by specific coagulopathy, such as fibrinogen deficiency, platelet dysfunction, or hyperfibrinolysis, and support timely and focused therapeutic intervention. By providing a functional summary of coagulation rather than fragmented static lab values, TEG and ROTEM make possible a precision medicine approach to transfusion and minimize utilization of blood products unnecessarily as well as its complications [112,113,116].

In tandem, point-of-care testing (POCT) augments intraoperative and critical care decision-making by providing rapid bedside access to key laboratory parameters. This comprises immediate hemoglobin and hematocrit measurements that guide transfusion thresholds and blood loss quantification. Arterial blood gas measurement is also invaluable, supplying real-time information about acid–base status, oxygenation status, and lactate status—parameters that indicate tissue perfusion and resuscitative adequacy. Electrolyte monitoring of particular interest is that of calcium, potassium, and magnesium to guide cardiac stability and functioning of coagulation cascade and to permit timely correction of derangements. Rapid coagulation studies turnover of a PT, INR, and aPTT also enables quick clinical decision-making in situations of active bleeding [116]. Continuous monitoring of hemodynamic and laboratory parameters is essential during high-risk spinal surgeries. Institutions should utilize point-of-care coagulation testing to facilitate rapid clinical decisions. Every MTP activation should be fully documented, with analysis of transfusion patterns and patient outcomes to ensure ongoing quality improvement [78].

### 7.5. Massive Transfusion Protocol Guidelines for Spine Surgery

MTP availability should be ensured for all high-risk spinal surgeries, including those involving more than four vertebral levels, three-column osteotomies, and spinal tumor resections [79,110]. TXA should be administered in accordance with established protocols, with consideration for higher dosing in complex deformity corrections based on institutional guidelines [20,21]. Autotransfusion techniques and topical hemostatic agents should be integrated into surgical workflows, supported by comprehensive team training and coordinated communication with the blood bank [107,115]. Discharge hemoglobin levels should be monitored closely, as low levels have been correlated with increased rates of hospital readmission. Correction of anemia should be planned before discharge [23]. Finally, institutions should establish clear transfusion protocols for patients who decline blood products, such as Jehovah’s Witnesses, undergoing complex spinal deformity surgery [117].

## 8. Postoperative and Long-Term Management

Postoperatively, patients must be monitored in an ICU setting to pick up early evidence of and manage promptly postoperative problems. Focused monitoring should involve serial hemoglobin and coagulation studies to detect trends that suggest continuing blood loss or coagulopathy. Drainage output must be attended to scrupulously for signs of active bleeding or delayed bleeding, and physicians must watch closely hemodynamic stability as well as signs of appropriate end-organ perfusion.

In those patients having ongoing coagulopathy or lab evidence of DIC-like phenomenon (disseminated intravascular coagulation), focused therapy could be required. This could consist of cryoprecipitate administration or fibrinogen concentrate administration or even prothrombin complex concentrate (PCC) administration in a directed fashion based on particular coagulation lab results and clinical symptomatology [6,54,116]. When pelvic bleeding is delayed postoperatively or clinical evidence of hemodynamic compromise is present, urgent re-exploration in a surgical setting is highly recommended to delineate and control hemorrhage sources. Furthermore, in those patients having pharmacologic anticoagulation contraindications or those having documented deep vein thrombosis (DVT) along with simultaneous bleeding, an inferior vena cava (IVC) filter placement could be required as a temporizing maneuver to provoke pulmonary embolism [72].

## 9. Patient Blood Management (PBM)

Patient blood management (PBM) is a systematic, multi-specialist approach that aims to reduce transfusion needs, improve perioperative results, and enhance patient protection. These three core pillars of PBM—preoperative optimization, intraoperative conservation, and enhancement of postoperative recovery—have been demonstrated to have important clinical effect.

### 9.1. Preoperative Optimization

Treatment of anemia is a keystone of PBM. Patients having hemoglobin < 12 g/dL must have thorough workup, selective therapy consisting of intravenous iron replacement, vitamin B12 or folate supplementation, and in some cases selected ones, erythropoiesis-stimulating agents. Preoperative anemia is found to elevate transfusion risk as well as result in adverse outcomes in varied spine surgery populations [1,61,62].

### 9.2. Strategies of Conservation During Surgery

Several intraoperative methods have been proven to be highly successful in decreasing the requirement for allogeneic blood transfusion in spine surgery. Antifibrinolytic therapy as a standard practice in spine surgery routines has been widely proven to be valid especially in high-risk spine surgeries. Level I evidence exists to demonstrate its effectiveness in substantially lowering intraoperative blood loss as well as reducing transfusion needs significantly, which has made it a standard of current blood management techniques [2,16,17,27,36,69,70,118].

Intraoperative cell salvage is a desirable method as well, particularly when undertaking long-segment spinal fusions or corrective deformity surgeries. These types of cases can significantly lower the recipient of donor blood products as well as provide hemodynamic stability [69,70,118].

Normovolemic hemodilution can also be used selectivity as a blood conservation measure. By decreasing circulation red cell concentration prior to expected blood loss, this method serves to reduce absolute loss of red cells during surgery.

Minimally invasive surgical (MIS) methods also play a big role in blood conservation during surgery. These methods have been proven to lessen muscle injury, reduce operative time, and minimize intraoperative bleeding—advantages that are most evident in spinal fusions that do not reset across fewer than three spine levels [2,30,37,40,44,104].

### 9.3. Postoperative Optimization

Restrictive blood transfusion thresholds that are tailored to patient comorbidities and oxygen delivery parameters are foundational to PBM. Optimized nutritional support, close observation of hemoglobin trends, and selective blood transfusion practice lower allogeneic product exposure further. Altogether, PBM programs have resulted in 20–40% declines in rates of blood transfusion and enhancements in short- and long-term results in complicated spine surgeries [60]. Table 2 discusses pillars of blood management of spine surgery.

## 10. Sequelae

Massive IBL has severe implications for perioperative results that go far beyond immediate hemodynamic instability into a delayed morbidity and even mortality.

Severe blood loss during surgery results in blood pressure drop, decreased tissue flow, and oxygen delivery impairment and generates a cascade of physiological distress that predisposes patients to severe consequences like acute kidney injury, myocardial ischemia, and cerebral hypoxia. These harmful effects are especially severe among older persons and those having a pre-existing cardiopulmonary disease due to a lower physiological reserve to cope up against hypovolemia and oxygen deficiency [1,4].

Perioperative neurological decline can be caused by a number of mechanisms. Nerve root compression can result from an enlarging hematoma or failure of hemostasis within the spinal canal. Hypotension during operation can lead to spasms of the spinal cord resulting in its ischemia injury when perioperative monitoring is lacking, especially in predisposed areas of poor blood supply. Excessive bleeding caused by coagulopathy can also lead to hematoma formation in the spinal canal causing compression of neural tissue. Early recognition of neurological signs and immediate re-exploration of operation theater is important to avert permanent neurological deficits and achieve optimal results [8,71].

Allogeneic blood transfusion entails a group of risks unrelated to the inherent complexity of surgery. These are transfusion-related acute lung injury (TRALI), febrile non-hemolytic or hemolytic reactions, and immunomodulatory consequences that predispose to postoperative infections. Serial transfusions predispose to iron overload and alloimmunization especially among patients treated in stages or undergoing revisions [39,72]. Additionally, excessive volume of transfusion will precipitate dilutional coagulopathy that will not only exacerbate ongoing hemorrhage but also expand the risk of reoperation from poor hemostasis [1].

Severe intraoperative blood loss and concomitant transfusions have been significantly correlated with a higher incidence of surgical site infections (SSIs). Evidence indicates that patients who receive more than four units of packed red blood cells (PRBCs) during surgery have virtually twice the risk of postoperative infection. Furthermore, long operative time and ongoing postoperative wounding drainage are independent risk factors for delayed epidermalization, dehiscence of the wound, and formation of seroma as risk factors leading to longer recovery and higher healthcare utilization.

Excessive intraoperative bleeding has been correlated with longer stays in the intensive care unit and overall hospital admission, as well as increased likelihood of unplanned reoperation. The physiological stress imposed by massive hemorrhage often necessitates extended monitoring and support, particularly in vulnerable patients. Notably, among individuals with an estimated blood loss exceeding 2000 mL, the 30-day postoperative mortality rate rises to 5.4%, compared to less than 1% in cases without such hemorrhage, underscoring the serious implications of uncontrolled intraoperative bleeding [22].

## 11. Prevention

Reduction in intraoperative blood loss (IBL) for spine surgery needs an individualized, proactive approach that commences a long time prior to surgery. Preoperative optimization directly involves attention to correcting anemia, which is among the most important modifiable risk factors. Every patient whose hemoglobin level falls below that of 12 g/dL must be treated specifically. Another important element of preoperative planning relates to anatomical assessment. Preemptive diagnosis of these risks can greatly reduce the likelihood of injury to a patient’s vessels and resultant bleeding throughout surgery. For highly vascular spinal neoplasms, preoperative arterial embolization has been extremely successful in patients. It consists of diagnostic angiography and selective tumor neovascularization embolization, and it has been demonstrated to significantly lower intraoperative bleeding and increase surgical field clarity. This enhances both safety and tumor resection time. Multidisciplinary communication is a second key to maximizing preoperative care. For difficult cases, especially in anterior lumbar interbody fusions (ALIF) surgery, a consultation from a vascular or general surgeon has been linked to reduced operative time, fewer problems, and much lower blood loss. This holds particularly in revision cases or complicated anatomical variation [1,24,119]. 

Along with these preoperative clinical steps, the risk factors which are for major intraoperative bleeding are anemia, deviated vascular anatomy, hyper-vascular tumors, reoperation, and spinal deformities which are in need of long-segment fusions. Identifying and avoiding these factors in the initial preoperative period can have a major impact on patient outcome [2,46,47,118]. To prevent extensive intraoperative blood loss (IBL) in spine surgery is necessary a proactive, personalized, and multifaceted approach that extends through all stages of patient care—preoperative, intraoperative, and postoperative. A combined approach not only lowers the requirement of blood transfusions and surgical morbidity but also contributes to improved recovery and long-term results.

## 12. Summary of Evidence on Reducing the Risk of IBL

Recommendations for reducing the risk of IBL include the following:

Perioperative individualized and comprehensive approach is important in reducing the risk of having a massive intraoperative blood loss especially in complicated spine surgeries like deformity corrections, tumor resections, and multilevel fusions of three or more levels of vertebral. Preoperative risk assessment is the initial important step. This is a complete anatomical and pathological review to spot those patients who are most risked to have a significant blood loss. These include past surgical history, vascularity of tumor, and severity of spinal deformity which all must be considered so that operative plan can be suitably made.

As a result of routine usage of antifibrinolytic therapy, particularly tranexamic acid (TXA), blood conservation is now a cornerstone. TXA must be given to candidate patients through intravenous and/or topical application in accordance with clinical context. Various randomized controlled studies and meta-analysis have proven that TXA lowers significantly the projected blood loss during surgery and requirement of blood transfusion without an elevation of thromboembolic events [7,9,10,11,14,16,18,21,23,27,69,70,71,97].

Just as important is anemia preoperative correction. Preoperative anemia patients should have hemoglobin levels above 12 g/dL. They should receive focused administration methods of hemoglobin improvement such as oral or intravenous iron replacement along with replacement of vitamin B12 or folate. Even selected application of erythropoiesis-stimulating agents has been practiced to prevent anemia. Proper anemia improvement has been correlated to lower rates of transfusion and better perioperative results [1,40,60,108,112,113,116].

In lumbar anterior procedures, especially L4–S1 anterior lumbar interbody fusion (ALIF) levels, co-operation with surgeons from vascular or retroperitoneal access is highly advocated. Such inter-professional co-operation is highly efficient in reducing vascular injury risk along with its hemorrhagic complication that subsequently occurs and by this means increases protection as well as effectiveness [71].

For high-volume blood losses that happen intraoperatively, early institution of intraoperative cell salvage and activation of a Massive Transfusion Protocol (MTP) is required. The MTP must be balanced-based resuscitation principles that provide packed red blood cells (PRBCs), fresh frozen plasma (FFP), and platelets in a 1:1:1 ratio. This practice must be driven by real-time viscoelastic coagulation monitoring tools including thromboelastography (TEG) or rotational thromboelastometry (ROTEM) that assist in guiding specific hemostatic therapies [6,54,116].

Last of all, perioperative vigilance is important in detecting continuing bleeding, coagulopathy or new neurological deficits. These will necessitate prompt recognition and intervention to avert conversion to irreversible or lethal consequences. Intensive monitoring, bolstered by serial laboratory studies and clinical observation, remains crucial to optimal recovery and patient protection.

This underscores the need for anticipating and managing massive blood loss with preparedness and skill [26,92].

While each of these strategies offers individual benefits, the most favorable outcomes are achieved through a comprehensive, multidisciplinary approach to blood management. Institutions should aim to implement evidence-based protocols tailored to their surgical population and resources.

## 13. Conclusions

Preemptive control of blood loss in spinal surgery involves a proactive individualized strategy initiated many hours prior to surgery. Correction of preoperative anemia is necessary, particularly in patients whose hemoglobin levels are lower than 12 g/dL. Pre-surgical anatomical evaluations identify the risks of vessels and avoid bleeding. Preoperative embolization has been successful in diminishing bleeding in highly vascular spinal hemangioblastomas. Cooperation with general or vascular surgeons decreases operative time, postoperative complications, and transfusion requirements. Determining risk factors such as anemia, tumor vascularity, and deformities of the spine is important in pre-planning. Tranexamic acid (TXA) decreases bleeding and transfusion requirement profoundly. It does not worsen clotting tendency. Intraoperative methods such as cell salvage and Massive Transfusion Protocols (MTPs) control bleeding. Real-time monitoring devices such as TEG or ROTEM direct focused replacement of blood. It has the best results when it is thorough, multidisciplinary, and evidence-informed.

## Figures and Tables

**Table 1:** Risk factors for massive intraoperative blood loss in spine surgery.

Patient-Related	Surgical Site	Anatomical Site	Associated with Medical Conditions
Demographics: Advanced age (>65 years) Hematologic: Preoperative anemia (Hb < 12 g/dL), coagulopathy, bleeding disorders Physical: Obesity (BMI > 30 g/m^2^), poor nutritional status Medications NSAIDs, platelet agents aspirin, clopidogrel), NOACs (dabigatran, rivaroxaban)	Complexity surgery: Multilevel instruction (>5 levels), combined anterior–posterior approaches Techniques: Osteotomies (PSO, VCR, SPO) Duration: Operative time > 4 h Staged procedures Approach: Anterior cervical approaches Revision Status: Scar tissue, altered anatomy, hardware removal	Vascular Proximity: Major vessel proximity (aorta, vena cava, vertebral arteries) Venous Anatomy: Dense epidural venous plexus, especially at thoracolumbar junction Bone Vascularity: Highly vascularized vertebral bodies Dural Considerations/Adherent dura	Tumors: Hypervascular metastases (renal, thyroid) Hemangiomas, vascular malformations Deformity: Severe scoliosis (>70 degrees Cobb angle) Kyphoscoliosis Bone Quality: metabolic bone Inflammatory: Ankylosing spondylitis, rheumatoid arthritis. Infectious Comorbidities: Liver disease, renal dysfunction, cardiovascular disease

**Table 2:** Pillars of blood management in spine surgery.

Patient Blood Management (PBM)	Surgical and Anesthetic Techniques	Tranexamic Acid (TXA)	Massive Transfusion
Anemia correction	MIS embolization,	antifibrinolytic	Protocol (MTP) 1:1:1 ratio
Restrictive transfusion	Hemostatic agents	decreasing blood loss 30–60%	viscoelastic guidance

## Data Availability

No new data were created or analyzed in this study. Data sharing is not applicable to this article.
